# Hematoma-induced Twiddler-like phenomenon as a presentation of DBS hardware failure: Case report

**DOI:** 10.3389/fnhum.2023.1160237

**Published:** 2023-04-21

**Authors:** Marshall T. Holland, Abraham Alvarado-Gonzalez, Joshua K. Wong, Leonardo Brito de Almeida, Aparna Wagle Shukla, Wissam Deeb, Addie Patterson, Michael S. Okun, Kelly D. Foote

**Affiliations:** ^1^Department of Neurosurgery, University of Alabama at Birmingham, Birmingham, AL, United States; ^2^Department of Neurology, Norman Fixel Institute for Neurological Diseases, University of Florida, Gainesville, FL, United States; ^3^Department of Neurosurgery, Norman Fixel Institute for Neurological Diseases, University of Florida, Gainesville, FL, United States; ^4^Department of Neurology, University of Minnesota, Minneapolis, MN, United States; ^5^Department of Neurology, University of Massachusetts, Worcester, MA, United States

**Keywords:** deep brain stimulation, Twiddler's syndrome, neurostimulator failure, pocket hematoma, impulse generator

## Abstract

Deep brain stimulators (DBS) may fail for a multitude of reasons. We present a 79-year-old Parkinson's disease patient who suffered a DBS failure following impulse generator (IPG) replacement surgery due to the IPG flipping within an expanded capsular pocket. This creation of the pocket was unintentional, and the pocket formed around an undiagnosed postoperative hemorrhage. The syndrome could be considered “Twiddler-like” because it resulted in device flipping. There were, however, many characteristic differences between our case and classical Twiddler's syndrome. DBS neurostimulator failure due to hematoma induced device flipping should be suspected when device interrogation is impossible or there are abnormally high impedances across multiple DBS lead contacts. A plain film X-ray series should be ordered and can be useful in providing radiological evidence of device flipping. In cases like ours the extensive braiding encountered in Twiddler's syndrome may be absent. Anchoring the IPG to a deep fascial layer as well as the use of an antimicrobial pouch are two methods that may be employed to prevent or to treat this complication.

## Introduction

A deep brain stimulator (DBS) device consists of an intracranially placed lead, a subcutaneous extension wire, and implantable pulse generator (IPG). The IPG is most commonly placed in the subclavicular location. A minority of patients will undergo IPG placement in an abdominal location. The clinician must be able to connect and to facilitate communication with the device through a handheld programmer in order to pursue device maintenance and programming. In the case of a rechargeable device, the patient must be able to connect to the device to enable the wireless charging function. If the device flips over, it may not be accessible for programming or for charging. We report an unusual case of neurostimulator device failure due to “flipping” over in an expanded subcutaneous capsular pocket that formed around an undiagnosed postoperative hematoma.

## Case report

A 79-year-old woman with advanced Parkinson's disease (PD) and BMI of 32.3 presented for troubleshooting of her DBS device. Her first symptom of PD was a right upper extremity tremor. As her disease progressed, she developed worsening tremor in both upper extremities, dystonia, and cramping of the right foot. She also progressed to develop motor fluctuations, severe bradykinesia, and dyskinesias. She did not have any significant psychiatric co-morbidities such as obsessive compulsive disorder. Significant dementia was not identified on neuropsychological screening and she was deemed a good candidate for DBS. The patient did not have any history of bleeding diathesis and was not taking any antiplatelet or anticoagulation medications. She underwent bilateral globus pallidus interna (GPi) DBS implantation without complication and the IPG (non-rechargeable) was placed in her abdomen which was her preference for cosmesis. During elective replacement of the IPG for battery depletion she requested placement of a rechargeable neurostimulator. Prior to replacement, the patient had not experienced any recent weight loss. A new, more superficial, pocket for the device was created to accommodate charging. The device was tested intraoperatively and found to have normal impedances and full wireless pairing with the external charging device was confirmed. The device was anchored to the underlying tissue with 2-0 silk at the two anchoring sites in the standard fashion.

**Figure 1:**
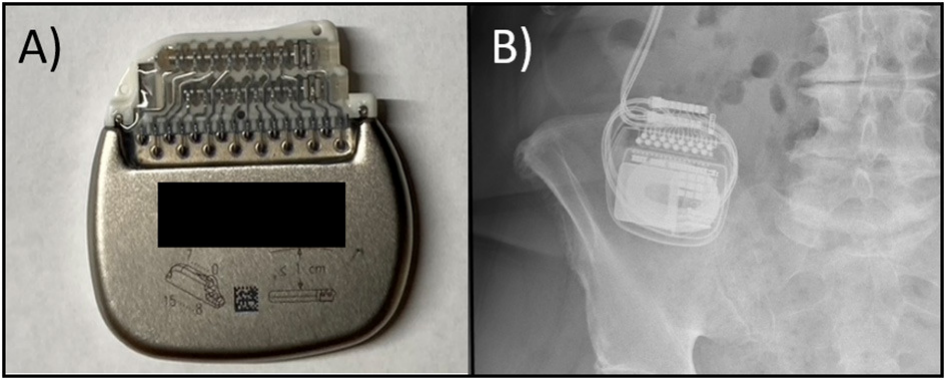
**(A)** Photo of a rechargeable IPG with the expected normal positioning of the DBS leads which exit to the right of the device; the device label is noted to have an upward orientation with the manufacturing label intentionally obscured, **(B)** A plain film X-ray series from our case demonstrating the abnormal insertion of the leads on the left side suggestive of flipping.

One month following the neurostimulator replacement surgery, the patient alerted the clinic that she had (for a week) been unable to communicate with or charge her device. During evaluation in clinic the device could not be interrogated, and programming was not possible. There were no abnormal physical exam findings. Plain film X-rays revealed the IPG had flipped 180° along its long axis (see Figure 1). Upon palpation of pulse generator site, we were unable to manually induce a flip of the device. The patient returned to the surgical suite that week where upon exploration of the IPG pocket, a large brown liquefied hematoma was observed. Following drainage, a large capsule was encountered. The IPG was restored to the correct orientation and replaced in a revised pocket, which was made smaller, and the IPG was anchored to the anterior abdominal wall with 2-0 silk at both anchoring sites on the IPG. Intraoperative interrogation of the device demonstrated normal functioning. Subsequent discussion with the patient revealed that there was swelling at the surgical site several days following the IPG replacement surgery, but denied any significant bruising. However, she did not seek care for the swelling until there was a failure to charge the device. Additionally, the patient could not recall any trauma to the abdomen or surgical site following the index operation prior to revision surgery. Six months after the intervention, the patient continued to be able to interrogate and charge her device and was receiving good therapeutic benefit from the system.

## Discussion

**Figure 2:**
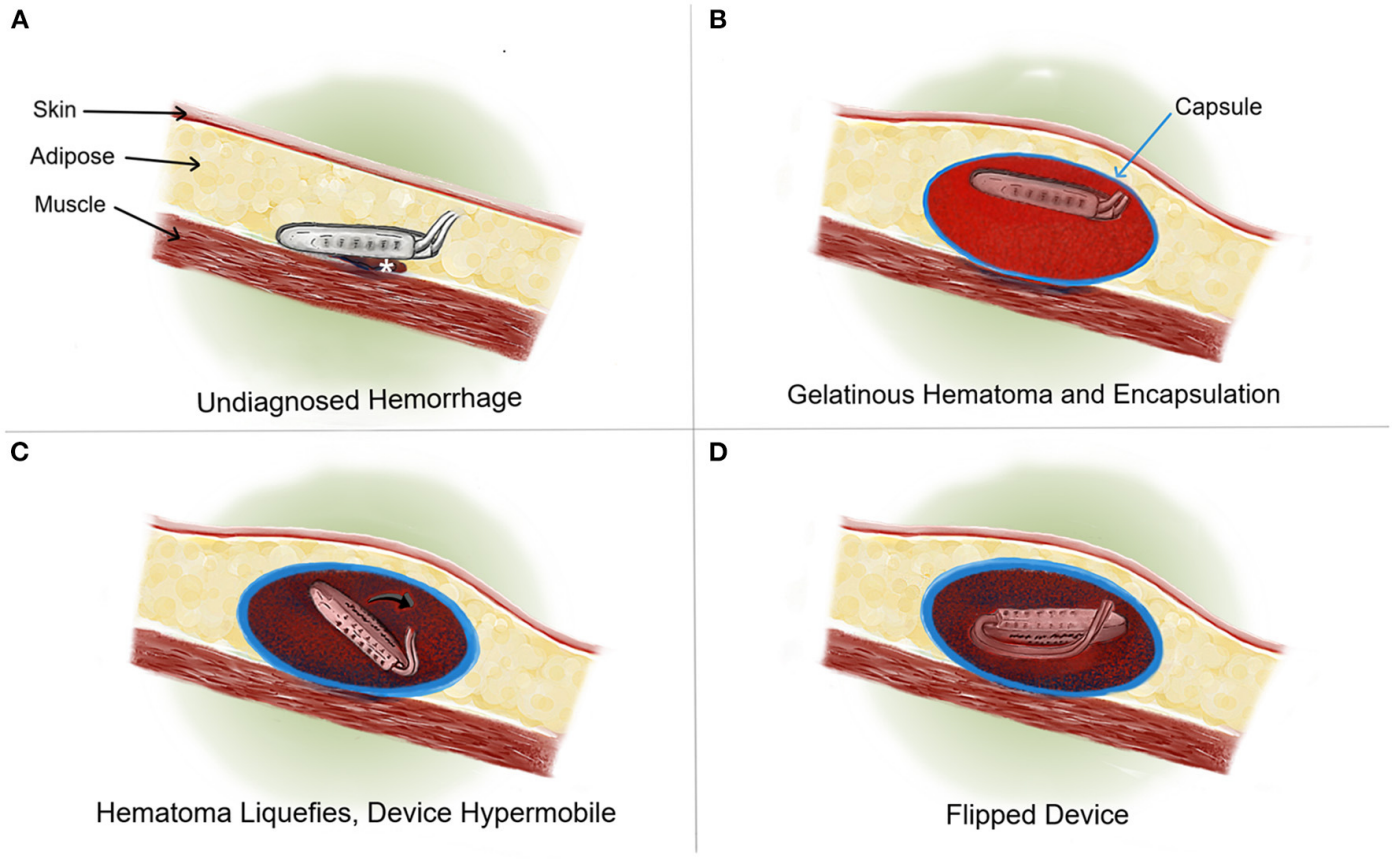
Cross sectional rostral to caudal view medical illustration. **(A)** An example of an IPG implanted with a resulting bleed (*). **(B)** Formation of a solid, gelatinous clot which expands until cessation of bleeding. A fibrous capsule is formed around the IPG and clot over the course of several weeks. At this time the IPG remains in appropriate orientation that facilitates interrogation and charging. **(C)** Over time, the blood clot evolves, breaking down into liquid blood. This scenario results in the hypermobility of the IPG (→) within a now enlarged and well-formed capsule. **(D)** The hypermobile IPG is “flipped” and cannot be charged by the patient or programmed by the clinician.

We observed a rare pocket hematoma resulting in neurostimulator (IPG) flipping with device failure. This complication manifested with an inability of the patient to recharge her device. We hypothesize that hemorrhage created an acute gelatinous hematoma that locked the IPG in a partially flipped position, facilitating formation of a fibrous capsule around the IPG and hematoma. The evolution into a chronic liquid hematoma likely allowed the IPG to mobilize within the enlarged capsule, rotate, and then become lodged in a suboptimal position for device communication, charging, and programming. Figure 2 provides a summary of the events likely leading to device failure. One could also speculate that the device became mobile following surgery, flipped, and initiated a slow bleed leading to a hematoma and enlarged pulse generator capsule. This would result in a similar situation of hypermobility, flipping of the device, and ultimately device failure due to an inability to charge or program the device once settled in the inappropriate position. Both the abdominal placement of the IPG and the patient's obese BMI of 32.3 put her at risk for this to occur (Burdick et al., 2010).

This phenomenon resembles Twiddler's syndrome (TS), however based on its characteristics it would not qualify for this diagnosis. First reported in 1968, shortly after the introduction of implantable pacemakers (Bayliss et al., 1968), TS is a rare complication caused by repeated flipping of the neurostimulator within the implanted pocket. Traditionally, TS flipping due to the unintentional manipulating or picking of the device (i.e., “twiddling”). The estimated TS prevalence in cardiac pacemakers is 1% of all implantation malfunctions (Hill, 1987). In neurostimulation, TS has been estimated to account for 1.3% of all DBS malfunctions (Burdick et al., 2010). TS has been demonstrated in the spinal cord stimulation (Son et al., 2018) and the vagal nerve stimulation literature (Trout et al., 2013). Risk factors for TS include surgical technique, unconscious flipping of the device by the patient (Menghetti et al., 2014), obese body habitus (Femenia et al., 2010), early return to exercise (Bracke et al., 2005), and the shape of the device (Gul et al., 2017). Obsessive compulsive symptoms may also contribute and the TS patient may not be conscious that they are flipping the device (Femenia et al., 2010; Moliz et al., 2015). It has been postulated that the abdominal pulse generator location, as was the location in our patient, may be more prone to flipping then the chest site (Boyle et al., 1998; Burdick et al., 2010; Gelabert-Gonzalez et al., 2010). Given the many potential reasons that could lead to device hypermobility, flipping, and failure, we believe the field should evaluate the use of the term TS to describe this phenomenon.

The repetitive flipping of the devices usually presents as a loss of benefit. It may lead to hardware failure, often associated with out-of-range elevated impedances discovered to be present across all electrode contacts (open-circuit), and potentially associated with an inability to communicate with or to charge the device. TS can result in lead fracture or migration. Although the flipping occurs in the pocket, the lead damage is often rostral to the IPG and may be in the nuchal region. Though our case may not be due to twiddling, as our patient denied any manipulation of the device after surgery, the result of the single flip in orientation due to a hematoma resulted in Twiddler-like manifestations.

The workup for device flipping or TS includes interrogation of the device, specifically searching for an open or short circuit, as well as obtaining plain film X-rays. The imaging usually reveals an inappropriate position of the neurostimulator that “flipped” along the long axis (see Figure 1). The electrical leads may be dislodged or displaced and there may possibly be twisting or braiding of the extension wire although any braiding or twisting is usually minimal as the device usually flips only once and at the rotation is 180 degrees or less. Prevention and treatment are similar with the goal of firmly securing the neurostimulator within the device pocket. Anchoring the IPG to a strong fascial layer with a non-absorbable suture has been recommended (Sobstyl et al., 2017). An antimicrobial pouch can also be used to decrease IPG mobility (Osoro et al., 2018). The pouch is thought to manifest its effectiveness by occupying more space in the pocket, and thus inducing a more robust inflammatory response. It may also work through increasing friction between the IPG-surface and the surrounding tissue (Shandling et al., 1991; Osoro et al., 2018).

This report demonstrates a previously undescribed and unusual presentation of device failure due to a postoperative implantation hematoma and expanded capsule. Prior literature demonstrates that all neurostimulator systems retain the risk for device flipping or patient twiddling leading to device failure. A strength of this report is our presentation of mitigation techniques to prevent this complication. While we are limited to our timepoints of evaluation in this case, as we did not have the opportunity to evaluate the patient when she noted the swelling, we were able to observe the consequences of this with the expanded device capsule.

## Conclusion

In conclusion, we report a novel cause for IPG flipping. It is unknown whether this complication will be more common in the abdominal or subclavicular IPG location and future reports may help to clarify this point. Utilizing a technique to reduce mobility of the neurostimulator may reduce this potential complication.

## Data availability statement

The original contributions presented in the study are included in the article/Supplementary material, further inquiries can be directed to the corresponding author.

## Ethics statement

Ethical review and approval was not required for the study on human participants in accordance with the local legislation and institutional requirements. The patients/participants provided their written informed consent to participate in this study. Written informed consent was obtained from the individual(s) for the publication of any potentially identifiable images or data included in this article.

## Author contributions

MH, AA-G, MO, and KF conceptualized the study. MH and AA-G gathered the data. MH wrote the first draft of the study. All authors provided crucial input about the study, critically evaluated, edited, and approved the final version of the manuscript.
