# Supplementary material for: Hematoma-induced Twiddler-like phenomenon as a presentation of DBS hardware failure: Case report
Source: Front Hum Neurosci. 2023 Apr 21;17:1160237. doi: 10.3389/fnhum.2023.1160237 (PMC10161199; doi:10.3389/fnhum.2023.1160237)
Supplement: Supplementary file 1 [file Data_Sheet_1.PDF]

CARE case report guidelines

CARE Checklist of information to include when writing a case report

(cc) BY-NC-ND

Topic	Item	Checklist item description	Reported on Line
Title	1	The diagnosis or intervention of primary focus followed by the words "case report"	Title Page: Page 1, Lines 1-2
Key Words	2	2 to 5 key words that identify diagnoses or interventions in this case report, including "case report"	Title Page: Page 1, Lines 50-51
Abstract (no references)	3a	Introduction: What is unique about this case and what does it add to the scientific literature?	Main Document, Page 2, Lines 25-27
	3b	Main symptoms and/or important clinical findings	Main Document, Page 2, Lines 14-17, 24-26; Page 3, Lines 5-7
	3c	The main diagnoses, therapeutic interventions, and outcomes	Main Document, Page 3, Lines 1-4, 7-9
	3d	Conclusion—What is the main "take-away" lesson(s) from this case?	Main Document, Page 4, Lines 13-23
Introduction	4	One or two paragraphs summarizing why this case is unique (**may include references**)	Main Document, Page 3, Lines 12-18
Patient Information	5a	De-identified patient specific information.	Main Document, Page 2, Lines 14-22
	5b	Primary concerns and symptoms of the patient.	Main Document, Page 2, Lines 24-25, Page 3, Lines 5-6
	5c	Medical, family, and psycho-social history including relevant genetic information	Main Document, Page 2, Lines 14-17
	5d	Relevant past interventions with outcomes	Main Document, Page 2, Lines 17-22
Clinical Findings	6	Describe significant physical examination (PE) and important clinical findings.	Main Document, Page 2, Lines 25-26; Page 3, Line 1
Timeline	7	Historical and current information from this episode of care organized as a timeline	Main Document, Page 2, Lines 24-26; Page 3, Lines 1-9
Diagnostic Assessment	8a	Diagnostic testing (such as PE, laboratory testing, imaging, surveys).	Main Document, Page 2, Lines 25-26; Page 3, Line 1, Figure 1
	8b	Diagnostic challenges (such as access to testing, financial, or cultural)	Main Document, Page 3, Lines 6-8
	8c	Diagnosis (including other diagnoses considered)	Main Document, Page 3, Lines 1-3, Page 4, Line 12
	8d	Prognosis (such as staging in oncology) where applicable	Not Applicable
Therapeutic Intervention	9a	Types of therapeutic intervention (such as pharmacologic, surgical, preventive, self-care)	Main Document, Page 3, Lines 1-6
	9b	Administration of therapeutic intervention (such as dosage, strength, duration)	Not Applicable
	9c	Changes in therapeutic intervention (with rationale)	Not Applicable
Follow-up and Outcomes	10a	Clinician and patient-assessed outcomes (if available)	Main Document, Page 3, Lines 8-9
	10b	Important follow-up diagnostic and other test results	Not Applicable
	10c	Intervention adherence and tolerability (How was this assessed?)	Main Document, Page 3, Lines 8-9
	10d	Adverse and unanticipated events	None
Discussion	11a	A scientific discussion of the strengths AND limitations associated with this case report	Main Document, Page 5, Lines 1-7
	11b	Discussion of the relevant medical literature **with references**.	Main Document, Page 3, Lines 20-26; Page 4, Lines 1-23
	11c	The scientific rationale for any conclusions (including assessment of possible causes)	Main Document, Page 3, Lines 12-18
	11d	The primary "take-away" lessons of this case report (without references) in a one paragraph conclusion	Main Document, Page 5, Lines ***
Patient Perspective	12	The patient should share their perspective in one to two paragraphs on the treatment(s) they received	Unable to reach patient
Informed Consent	13	Did the patient give informed consent? Please provide if requested	Yes ☒ No ☐
